# Bulbocavernosus Reflex Test for Diagnosis of Pudendal Nerve Injury in Female Patients with Diabetic Neurogenic Bladder

**DOI:** 10.14336/AD.2016.0309

**Published:** 2016-12-01

**Authors:** Xiaoting Niu, Xun Wang, Huanjie Huang, Peiqi Ni, Yuanshao Lin, Bei Shao

**Affiliations:** Department of Neurology, the First Affiliated Hospital, Wenzhou Medical University, Wenzhou 325000, Zhejiang, China

**Keywords:** diabetic neurogenic bladder (DNB), bulbocavernosus reflex (BCR), nerve conduction study (NCS), female, diagnosis

## Abstract

The study was designed to investigate the clinical application and significance of the bulbocavernosus reflex (BCR) test for diagnosing diabetic neurogenic bladder (DNB) in female subjects. In this study, 68 female patients with DNB and 40 female normal controls were subjected to a nerve conduction study (NCS) of all four limbs and the BCR test. The data were analyzed and compared, and the corresponding diagnostic sensitivities were discussed. Mean BCR latency for female DNB patients was significantly prolonged, compared to that of the control group, suggesting pudendal nerve injuries in female DNB patients. Moreover, DNB patients were categorized according to the diabetes course. Compared to that of Group A (diabetes course < 5 y), the mean BCR latency was significantly prolonged in Group B (diabetes course between 5 and 10 y) and then further prolonged in Group C (diabetes course > 10 y), which were all longer than the control group. Furthermore, compared with that of the controls, the mean BCR latency was prolonged in DNB patients with or without NCS abnormalities in limbs. Nevertheless, no significant difference was observed in BCR latency between DNB patients with and without NCS abnormalities. Significantly increasing trends were also observed in the NCS and BCR abnormality rates along with increased diabetes course. Most importantly, compared with the NCS of limbs, the BCR test was more sensitive in diagnosing DNB in the female subjects. Overall, our findings suggest that the BCR test would help to assess the pudendal nerve injury in female DNB patients, which might be a potential diagnostic tool in the clinic.

In China, the adult diabetes prevalence rate is approximately 9.7% in 2008 [1], which has become a major public health problem. Diabetic neurogenic bladder (DNB) is defined as a variety of urinary bladder dysfunction exclusively induced by diabetes, and is mainly characterized by pudendal neuropathy in the urinary tract. It has been reported that the incidence of DNB is between 40% and 80% [2]. Even with good control of blood glucose, the disease incidence would be around 25% [3-4]. Moreover, the incidence of sphincter dysfunction is about 25% in patients suffering from diabetes for over 10 years, which would increase to more than 50% after 15 years [5]. Therefore, higher DNB incidence has been revealed in diabetic patients, which significantly influences the life quality of these patients.

DNB has been shown to be closely associated with peripheral neuropathy in diabetes [6], and peripheral neuropathy could be observed in 75% to 100% of patients with bladder lesions. Currently, nerve conduction studies (NCS) of all four limbs are widely used in the clinical detection of peripheral neuropathy in diabetes. However, NCS cannot provide a valid diagnosis on pudendal neuropathy. Therefore, it is of great importance to develop a reliable and objective detection method for pudendal neuropathy in DNB, especially for early DNB patients without limb-nerve injuries. Bulbocavernosus reflex (BCR) refers to the contraction of bulbocavernosus muscle induced by stimulating the pudendal nerves. BCR is conducted along the sacral reflex arc, whose latency reflects the integrity of the surrounding afferent nerves, sacral cord, and efferent motor fibers. Therefore, BCR would contribute to the assessment of pudendal neuropathy in DNB. It has been shown that BCR could help to assess pudendal neuropathy accompanied by sphincter dysfunction [7-9]. Further, dramatically prolonged BCR latency has also been observed in the DNB patients [10].

The present study was designed to evaluate the clinical application and significance of BCR test in diagnosing DNB in female patients. Female DNB patients were subjected to the NCS and BCR tests, and the diagnostic sensitivities of these detection methods were analyzed and discussed.

## MATERIALS AND METHODS

### Study subjects

68 female patients with DNB (average age of 52.15 ± 6.87 y; ranging over 43-70 y) were admitted to the First Affiliated Hospital of Wenzhou Medical University from January 2009 to January 2014. DNB was diagnosed according to the following criteria [11]: (1) diabetes in line with the diagnostic criteria from the WHO (1999); (2) clinical symptoms included frequent urination, urgent micturition, urinary weakness, urine dripping, prolonged urination or incontinence, and difficulty urinating; (3) residual urine > 100 mL detected by B ultrasound; (4) urinary retention, except induced by urinary stones, tumors, and trauma.

According to the NCS of all four limbs, these DNB patients could be divided into the normal NCS group (26 patients, with an average age of 51.65 ± 7.01 y, ranging over 43-68 y) and the abnormal NCS group (42 patients, with an average age of 52.99 ± 6.30 y, ranging over 45-70 y). Moreover, based on the disease course of diabetes, the patients were divided into the following groups: (1) Group A with disease course < 5 y, which included 21 DNB patients (average age of 51.74 ± 7.06 y; ranging over 43 - 69 y); (2) Group B with disease course between 5 and 10 y, which included 27 DNB patients, (average age of 52.62 ± 6.59 y; ranging over 44-70 y); and (3) Group C with disease course > 10 y, which included 20 DNB patients (average age of 51.96±6.82 y; ranging over 44 - 70 y). In addition, 40 healthy female subjects were included as controls (average age of 52.81 ± 7.73 y; ranging over 42 - 74 y). Prior written and informed consent were obtained from every patient and the study was approved by the ethics review board of the First Affiliated Hospital of Wenzhou Medical University.

### Nerve Conduction Study of all four limbs

NCS of all four limbs was performed with the Keypoint EMG/evoked potential instrument (Denmark). Skin temperature was maintained at 30-32°C during detection. The test covered motor and sensory nerve fiber latency, amplitude, and conduction velocity of the median and ulnar nerves in both upper limbs, as well as the peroneal and tibial nerves in both lower limbs.

The subject was kept in a supine position, with the ground electrode placed between the stimulating and recording electrodes. The results of limb nerve conduction were assessed according to the criteria that were established by the Neurophysiological Laboratory at the Beijing Union Medical College Hospital (Beijing, China). Neuropathy was indicated by the prolonged latency, decreased amplitude, and declined motor conduction velocity (MCV) or sensory conduction velocity (SCV).

For motor conduction determination, the stimulating electrode was placed in the neural stem, and the recording electrode was placed in the muscle belly. The reference electrode was placed in the surrounding tendon or attachment points. The stimulating intensity was set at 10% to 30% more than the intensity that could induce the largest muscle action potential. Stimulating time was set as 0.1ms. The latency was calculated from the baseline waveform, and the amplitude was determined based on the peak value. MCV was defined as the distance between the two different stimulating points at the proximal and distal ends of the neural stem divided by the difference in the reflex latency.

For retrograde sensory conduction detection, the recording electrode was placed in the end of the finger or toe. The fixation of stimulating electrode and the calculation of latency and amplitude were the same with the motor conduction detection. SCV was defined as the distance between the stimulating and recording electrodes divided by the evoking latency.

### BCR test

BCR test was performed with the same Keypoint EMG/evoked potential instrument. The subject was kept in a lithotomy position with the ground electrode placed at the ankle and the stimulating electrode placed at the pubic symphysis. The concentric needle recording electrode was sequentially inserted into the right and left bulbospongiosus muscles. The square-wave stimulation intensity was seven times that of the sensory threshold with stimulation frequency of 1.9 Hz. 20 reflex waves were recorded in total, and the average value was obtained. The latency was calculated from the baseline waveform. Scanning time was set as 5ms/div, and the analysis time was 100 ms, with the bandwidth of 10-2000 Hz.

### Statistical analysis

All data were expressed as mean ± SD. SPSS 16.0 software was used for statistical analysis. Analysis of variance and χ^2^ tests were used for comparison. *P* < 0.05 was considered as statistically significant.

**Figure 1.**
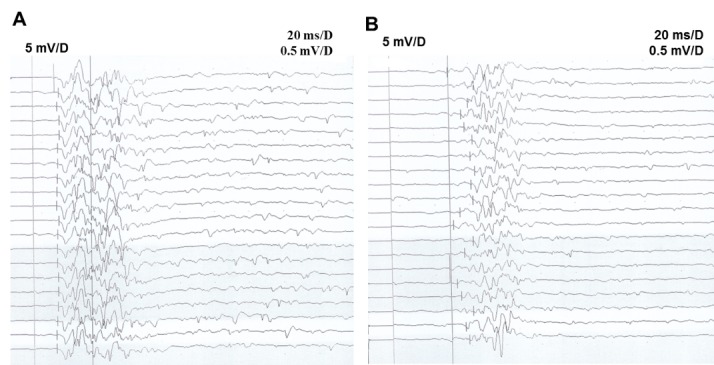
**BCR test results on representative subjects**. (**A**) A 50-year-old healthy woman. Stimulating electrode on the pudendal nerve and recording electrode in the left bulbocavernous muscle, with average BCR latency of 28.8ms, illustrating normal BCR. (**B**) A 51-year-old woman with DNB. Stimulating electrode on the pudendal nerve and recording electrode in the left bulbocavernous muscle, with average BCR latency of 62.8ms, illustrating prolonged BCR latency.

## RESULTS

### Abnormal BCR in female patients with DNB

The results from the BCR test were first analyzed by comparing female DNB patients and normal controls (Fig. 1). The BCR latency in the female DNB patients, with a group mean ± SD of 58.12 ± 6.30 ms, was significantly prolonged (*P* < 0.05; Table 1), compared to 44.34 ± 5.21ms in the control group. Next, BCR abnormalities in female DNB patients were compared between different diabetes course groups. Compared with Group A (diabetes course < 5 y), the mean BCR latency was significantly prolonged in Group B (diabetes course between 5 and 10 y), and further prolonged in Group C (diabetes course > 10 y) (both *P* < 0.05). All the mean BCR latencies in these three groups were longer than that of the control group (all *P* < 0.05; Table 1). Taken together, our results indicated that, BCR latency was significantly prolonged in female DNB patients (suggesting pudendal nerve injuries) as the length of the diabetes course increased.

### Association of NCS and BCR abnormalities in female DNB patients

NCS abnormalities in limbs mainly contained prolonged action potential latency, decreased amplitude, and declined MCV or SCV. Our results for the NCS of all four limbs showed that out of these 68 female DNB patients, there were 42 patients with NCS abnormalities and 26 patients that did not have any abnormalities. Compared with the control group, the BCR latency was prolonged in DNB patients with or without NCS abnormalities in limbs (*P* < 0.05). However, no significant differences were observed in BCR latency between the DNB groups with and without NCS abnormalities (*P* > 0.05; Table 2). These results suggest that abnormal BCR could be detected in female DNB patients irrespective of the presence of NCS abnormalities. Thus, we speculated that the pudendal nerve injuries in female DNB patients might occur before limb-nerve injuries.

**Table 1:** BCR latencies in female DNB patients and control subjects.

	N	Left BCR latency, ms	Right BCR latency, ms	Mean BCR latency, ms
Group A	21	50.49±6.99^*^	51.15±6.73^*^	51.07±6.89^*^
Group B	27	59.06±6.29^*^^#^	58.63±6.17^*^^#^	58.84±6.22^*^^#^
Group C	20	67.78±5.96^*^^#^^$^	67.20±6.14^*^^#^^$^	67.31±6.05^*^^#^^$^
Control	40	43.74±5.19	44.44±5.37	44.34±5.21

Note: compared with the control group,

* *P* < 0.05; compared with Group A,

# *P* < 0.05; compared with Group B,

$ *P* < 0.05.

**Table 2:** BCR latencies in DNB patients with and without NCS abnormalities.

	n	Left BCR latency, ms	Right BCR latency, ms	Mean BCR latency, ms
DNB patients without NCS abnormalities	26	57.78 ± 6.33^*^	58.31 ± 6.96^*^	58.04 ± 6.72^*^
DNB patients with NCS abnormalities	42	57.96 ± 6.42^*^^#^	58.95 ± 6.01^*^^#^	58.77 ± 6.09^*^^#^
Control	40	43.74 ± 5.19	44.44 ± 5.37	44.34 ± 5.21

Note: compared with the control group,

* *P* < 0.05; compared with DNB patients without NCS abnormalities,

# *P* < 0.05.

### Association of NCS/BCR abnormality rates and diabetes course in DNB

We then investigated a potential association between the NCS/BCR abnormality rates and diabetes course in these female DNB patients. The results showed that the NCS abnormality rates for Groups A, B, and C were 47.62%, 62.96%, and 75.00%, respectively (*P* < 0.05; Table 3), indicating that NCS abnormality rates were increased along with the increasing diabetes course. On the other hand, when BCR latency > 57.78 ms (calculated as the average BCR latency in the control group 44.34 ms + 2.58 SD) or showing no changed waveform was regarded as abnormal, the BCR abnormality rates for Groups A, B, and C were 71.43%, 83.33%, and 97.50%, respectively (*P* < 0.05; Table 3). Moreover, compared with the NCS of all four limbs, the BCR test was more sensitive in diagnosing DNB in these female patients (Table 3). Taken together, the BCR abnormality rate was significantly elevated along with the increasing diabetes course in female DNB patients with superior diagnostic sensitivity, compared with NCS.

## DISCUSSION

According to neurophysiological principles, the BCR test could be used to record the conduction time of pudendal nerves and the conduction function of the sacral reflex arc. In our previous studies, the BCR test has already been shown to be a highly sensitive and specific detection method for the diagnosis of nervous system diseases in adult male and female populations in China [12-15]. Normal BCR performance has also been established for Chinese male and female subjects, with significant differences in BCR latency between the sexes [16, 17]. Therefore, only female DNB patients were included in this study, and the results from the BCR test were analyzed and compared.

It has also been shown that in DNB patients, abnormal BCR is associated with ultrasound urodynamics, which both contribute to disease diagnosis [18]. However, urodynamics assessment is susceptible to the subjective factors in the disease diagnosis, while the BCR test is more reliable and objective. Moreover, Komeda*et al.* [19] also found that compared with normal subjects, the BCR latency is significantly prolonged in DNB patients. Furthermore, Rapidi *et al.* [20] also reported that based on 26 patients with diabetes (15 males and 11 females), no significant differences were observed in the BCR results between these diabetic patients with and without sphincter dysfunction. However, significant differences were observed when the sample size was increased three times. In this study, 68 female patients with DNB were subjected to the BCR test. Consistent with the above findings, our results showed that the BCR latency in DNB patients was significantly longer than the control group, indicating pudendal nerve injuries with varying degrees in these female DNB patients, which might be valuable for the localization and qualitative diagnosis of DNB. In this study, the female DNB patients were divided into three groups according to the disease course of diabetes. Our results showed that the BCR latencies in these groups were longer than the control group, and the BCR latency was prolonged along with the increasing diabetes course in female DNB patients. These results suggest that pudendal nerve injuries might be associated with the diabetes course in female DNB patients. In our previous studies, it has also been shown that the BCR abnormality rate for diabetic patients with DNB was higher than diabetic patients without DNB [13, 21]. Taken together, these findings suggest that BCR latency could be used to assess the severity of pudendal nerve injuries in DNB patients.

**Table 3:** NCS and BCR abnormality rats in female DNB patients and control subjects.

	N	NCS abnormality rate, n (%)	BCR abnormality rate, n (%)
Group A	21	10/21 (47.62%)^*^	15/21 (71.43%)^*^
Group B	27	17/27 (62.96%)^*^^#^	22/27 (81.48%)^*^^#^
Group C	20	15/20 (75.00%)^*^^#^^$^	19/20 (95.00%)^*^^#^^$^
Control	40	0	1/40(2.50%)

Note: compared with the control group,

* *P* < 0.05; compared with Group A,

# *P* < 0.05; compared with Group B,

$ *P* < 0.05.

You *et al.* [22] performed the NCS test on 14 diabetic patients with DNB and 20 diabetic patients without DNB. Their results indicated slower SCV and MCV in the DNB patients, with relatively longer nerve conduction latency. Based on these findings, DNB patients might be widely accompanied by peripheral neuropathy. In this study, we showed that compared with the control group, the mean BCR latency was prolonged in DNB patients with and without NCS abnormalities in all four limbs, while no significant differences were observed in the BCR latency between DNB groups with and without NCS abnormalities in all four limbs. Based on these findings, the pudendal nerve injuries might be occurring in advance of the limb-nerve injuries in these female DNB patients. In addition, our results showed that there were significant differences in the NCS and BCR abnormality rates between the DNB patients with different diabetes course. These results suggest that compared with the NCS of all four limbs, the BCR test would be more sensitive in diagnosing DNB in female subjects, which was specifically valuable for the early disease diagnosis in clinic.

In conclusion, the present study shows that pudendal nerve injuries in female DNB patients could be detected using the BCR test. BCR latency was closely associated with the diabetes course in the female DNB patients. Moreover, pudendal nerve injuries in female DNB patients might be occurring in advance of the limb-nerve injuries. Furthermore, the BCR test was more sensitive than the NCS of all four limbs in diagnosing DNB. Therefore, the BCR test might be a promising objective and accurate early disease diagnostic tool for DNB in female subjects in the clinic.
